# Gaps in the System: Supporting People Living With Dementia and Their Caregivers

**DOI:** 10.1093/geroni/igab046.2006

**Published:** 2021-12-17

**Authors:** Madeline King, Allie Peckham, Monika Roerig, Husayn Marani, Gregory Marchildon

**Affiliations:** 1 University of Toronto, Toronto, Ontario, Canada; 2 Arizona State University, Pheonix, Arizona, United States

## Abstract

As individuals are living longer, the prevalence of older adults living with dementia and other complex health and social care needs is on the rise (Alzheimer’s Association, 2020; CIHI, 2020). Correspondingly, efforts to develop supportive programming and policies for persons living with dementia (PLWDs) are of paramount importance (CIHR, 2019). The challenges faced by PLWDs and other complex health and social needs are widely known (CIHR, 2019), however, a systematic understanding of how and if current and long-standing efforts are adequately meeting the needs of these individuals remains elusive. This research sought to understand how program administrators, decision makers, PLWD, and caregivers across five North American jurisdictions (British Columbia, Ontario, Newfoundland and Labrador, New York State, and Vermont) perceived specific dementia care programs and support services within their respective jurisdictions. We performed an inductive analysis of semi-structured interviews (N=37) and identified on-going care gaps experienced by participants. We present three main gaps: 1) disconnected and uncoordinated system infrastructure, 2) lack of comprehensive services to meet the diverse needs of PLWD and their caregivers, and 3) inconsistency in how dementia is understood; with associated perceived remedies. The results suggest that even when attempts to address the needs of PLWD and their caregivers are put in place there remains significant limitations of systems. The perspectives of decision makers, program administrators and individuals with lived experience offer unique insight into how these experiences may be improved to better support the complex needs of PLWD and their caregivers.

